# Medical Statistics

**Published:** 1836-07

**Authors:** 


### MEDICAL STATISTICS.

### *Statistical Researches on Calculous Diseases.* By Dr. Civiale.

[M. Civiale having laid before the Academy of Sciences the voluminous results of his statistical labours on these diseases, M. Double was appointed to make the report. From this, very little valuable matter can be gleaned, as the reporter has occupied himself chiefly in pointing out what has not been done, and in discussing the question of the applicability of the "numerical method" to the elucidation of disease. M. Civiale appears to have collected above five thousand cases, treated by the most celebrated European surgeons of the present day, and his statements are founded on this solid basis. From a discussion which took place at the Academy, it appears probable that M. Civiale has not examined these cases in all their bearings, which may be the reason of the barrenness of M. Double's report. We shall extract the few conclusions which are given.]

As regards *the age* most subject to calculous complaints, it appears that more than half the sufferers are below fourteen years of age. At the hospital at Lyons, seven or eight children are operated on to one adult. This, however, is not true in all localities. Whilst the fact is most convincing in Wurtemburg, in the mountains of Lorraine and Barrois, among the Alps on the confines of Italy, in some counties in England, &c., yet, on the other hand, in very hot and very cold countries, adults and the old are more liable to the disease. Children with calculi are everywhere almost exclusively found amongst the poor; whilst adult and old patients are met with equally in different classes of society. Children are almost always exempt from genito-urinary diseases, which so cruelly torment the calculous patients of other ages.

M. Civiale finds the opinion that calculous diseases are most frequent in some countries and districts to be unsupported by his facts. Many causes lead to this error; *e. g.* the attention being particularly directed at any given place to the disease brings many cases from a distance. From the number of operations performed by the celebrated lithotomist Raw, in the hospital at Amsterdam, it was believed that the stone was very common in Holland; but since his death they have diminished one-third. The frequency of the return of the disease after lithotomy is confirmed. This is particularly the case where there is at the same time chronic catarrhus vesicæ, or any other confirmed disease of the bladder or prostate. M. Civiale *asserts* that such diseases cease more quickly, and in a more marked manner, after lithotrity than after lithotomy. The presence of the stone may produce two opposite states of the bladder: hypertrophy with diminution of capacity, or thinness of its coats with dilatation. Of 5,715 cases in which lithotomy had been employed by the first surgeons of Europe, there were 1,141 deaths, 4,478 complete cures, and 100 consecutive diseases. The mortality on the whole number for all ages was therefore a fifth; and it should be recollected that half these patients were under fifteen years, and at this age there is at least twice as great a chance of cure. On the other hand, in 257 cases of lithotrity collected in M. Civiale's tables, there were only six deaths, or a mortality of one in forty-two patients; and of the whole number there were only two or three individuals under fourteen years of age. As a further proof of the superiority of lithotrity, M. Civiale states that, since its discovery, among many medical men who have been affected with stone, it would be difficult to find one who had not preferred lithotrity to the old operation.

[This comparison of the average of the mortality of the two operations is probably as just as could be drawn from any available materials; but these are necessarily too defective to admit such sweeping conclusions as seem at first sight legitimate. Lithotrity is a new operation, and consequently its supporters would publish every successful case, and would choose generally those cases only in which there was a fair prospect of success. On the other hand, except in hospitals, where accurate tables of operations and their results are kept, (and they are comparatively few,) the rare, difficult, and often fatal cases of lithotomy are those which, from offering the greatest interest and instruction, would frequently be alone published; and very desperate cases are constantly operated on by the old method, as a last resource. The disproportion also of the numbers of the two tables (5,715 with 257,) is another ground of suspicion as to the value of the comparison. We know of more than one surgeon who was successful in his first thirty or forty cases of lithotomy, but whose average was speedily reduced by a few subsequent cases which terminated fatally. It may be doubted also whether the *personal* preference which medical men give to lithotrity is of much weight. The prospect at least of less immediate suffering, and an escape from the knife, are likely to be preponderating influences when a man is called on to decide for himself as to the particular operation which is to be performed on his own body; and neither of these reasons are the most important ones. The question, however, of the greatest practical value is not as to which operation is most fatal, but to what kind of cases lithotrity is most suited, and under what circumstances lithotomy is indispensable. The solution of these points M. Civiale has not yet attempted.]

*Gazette Médicale de Paris,* No. 42, 17 *Octobre,* 1835.

---

*On Suicide in the Canton of Geneva.* By M. PREVOST.

[BY the laws of the canton, each case of violent death is investigated by a police magistrate, and the documents are sent to the "procureur generale," and carefully preserved. M. Prevost has examined these documents, collected between 1825 and 1834 inclusively, with a view to investigating the causes of suicide, and of diminishing them, if possible. The following are the more important results.]

1. *Age.*

| Ages. | Number of Cases in 10 years. | Men. | Women. |
|---|---|---|---|
| From 50 to 60 | 34 | 25 | 9 |
| 20 to 30 | 30 | 22 | 8 |
| 60 to 70 | 19 | 10 | 9 |
| 30 to 40 | 18 | 15 | 3 |
| 40 to 50 | 15 | 13 | 2 |
| 70 to 80 | 9 | 6 | 3 |
| 10 to 20 | 5 | 3 | 2 |
| 80 to 90 | 3 | 1 | 2 |

From this table it appears that suicides are most frequent between 50 and 60 years of age. The age when the passions are the strongest (from 20 to 30) is, as might be expected, high in the scale; that of youth and old age low, from the young being strangers to the cares of life, and the old few in number when compared with the population.

2. *Sex, and State of Marriage or Celibacy.*

There are more suicides among men than women, in the proportion of 95 to 38, or about three to one; and more unmarried than married, or in the state of widowhood, in the proportion of 70 to 63, or about seven to six. Notwithstanding this, the female suicides are more numerous among the married and widows than among the unmarried, in the proportion of 21 to 17. But among men the proportions are reversed, that is, 42 to 53; so that, on the whole, suicides are more frequent among the unmarried than amongst those who are or have been married. This will not surprise those who know the energy, courage, and patience of women under misfortune; men more readily giving way to despair, and to vices consequent upon it. Men also have means of destruction, as fire-arms, more readily at hand.

### 3. *Occupations.*

The number of suicides are in proportion to the numbers of the individuals engaged in various trades, except among the agricultural population, where the proportion is very small. Thus, the agricultural population of the canton is 18,000, among whom, during ten years, there have been but twelve suicides; whereas, if they had been in the same proportion to the whole number as was found in the other occupations, they would have amounted to thirty-nine. Constant occupation, and hard yet healthy work, render them less sensible to the cares of life. There is also a somewhat larger proportion of suicides among the educated classes, who are engaged in literary pursuits or the higher branches of commerce.

### 4. *Religion.*

The relative proportion of Protestants to Catholics in the canton of Geneva is, according to the census of 1834, as 77 to 56. Thus,

| Of 133 inhabitants, there are | | Of 133 cases of suicide, |
|---|---|---|
| Protestants | 77 | 107 |
| Catholics | 56 | 26 |
| | 133 | 133 |

This result should attract the attention of those who are interested in the moral and religious education of Protestants.

### 5. *Means of Destruction.*

| | |
|---|---|
| Drowning | 55 |
| Fire-arms | 31 |
| Strangulation | 18 |
| Voluntary falls | 15 |
| Cutting instruments | 7 |
| Poison | 7 |
| | 133 |

In a small province with a lake and two rapid rivers, it is not surprising that drowning should be the most frequent mode of suicide; next to this is death by fire-arms, which is accounted for by all the men having fire-arms, as they are in the militia. Whilst the men have used fire-arms and cutting instruments, the women have almost alone had recourse to poisons and voluntary falls.

### 6. *Seasons.*

The seasons influence sensibly the number of suicides. There are more almost constantly in April.

Of 133 suicides, there were in

| | | | |
|---|---|---|---|
| April | 19 | March | 10 |
| June | 17 | November | 9 |
| August | 17 | September | 6 |
| July | 15 | January | 5 |
| October | 14 | February | 5 |
| May | 13 | December | 3 |

The spring appears to have an unfavorable effect, and during the great heats there are more suicides than during the cold weather. It is curious that many suicides happened on the same day or week. Thus, on April 9th, 1830, there were two suicides, and several others on the previous and subsequent days; on the 20th May, 1830, there were two suicides; on the 28th and 29th of March, 1831, two; and the same on the 3d and 4th of July of the same year. On April 20, 1833, there were two; and, on July 5th, 1833, two others. Some atmospheric changes may account for this, though meteorological tables did not satisfactorily explain them.

### 7. *Presumed Motives.*

| | | | |
|---|---|---|---|
| Physical disease | 34 | Bad conduct. Drunkenness | 10 |
| Insanity | 24 | Fear of punishment. Remorse | 6 |
| Losses of property | 19 | Disappointment in love | 6 |
| Domestic grief | 15 | Gambling | 4 |
| Melancholy without known cause | 13 | Mysticism | 2 |

### 8. *Relation of Suicides to the Population and to the Deaths.*

The number of suicides is, as to the whole number of deaths, as one to $90\frac{1}{8}$, and to the whole population as one to 3,985; the mean population of the canton during the last ten years being 53,0000.

| | | |
|---|---|---|
| In 1825 | 6 | suicides. |
| 1826 | 6 | — |
| 1827 | 9 | — |
| 1828 | 13 | — |
| 1829 | 13 | — |
| 1830 | 16 | — |
| 1831 | 18 | — |
| 1832 | 12 | — |
| 1833 | 24 | — |
| 1834 | 16 | — |
| | 133 | |

From this table it appears that the number of suicides has gradually increased from 6 as high as 24 in one year. The last year it decreased to 16; and it is fervently hoped that this deduction may be maintained, and that the increase may not be so frightfully rapid as it appears to have been. It must however be taken into account, that the population was, in 1822, 51,113, and, in 1834, 56,655. The police also are more active, and inquests are held more regularly.

(Extracted from the "Bibliothèque Universelle." *Genève.* Juin, 1835.)

*Annales d'Hygiène publique.* *Janvier,* 1836. No. 29.

---

### *The Cholera in Italy.*

*Statistical Report of the Cholera in Italy, in the year* 1835; *containing the approximative Results, from its invasion to its cessation, in the Cities where it has chiefly prevailed.*

| Places. | Total Population | Total Number attacked. | Total Deaths. | Proportion per cent. of the Population attacked | of Deaths in those attacked | Deaths to the whole Population. | Duration of the Epidemic. |
|---|---|---|---|---|---|---|---|
| Nice . . | 26,000 | 396 | 224 | $1\frac{1}{2}$ | $6\frac{2}{3}$ | $0\frac{9}{10}$ | 13 July—27 Sept. |
| Cuneo . . | 18,000 | 1,121 | 425 | $6\frac{1}{4}$ | 38 | $2\frac{1}{3}$ | 25 July—6 Sept. |
| Genoa . . | 80,000 | 4,259 | 2,151 | $5\frac{1}{3}$ | $50\frac{1}{2}$ | $2\frac{3}{4}$ | 1 August—13 Oct. |
| Leghorn . | 66,000 | 2,031 | 1,146 | 3 | $56\frac{2}{5}$ | $1\frac{3}{4}$ | 6 August—13 Oct. |

*Bullettino delle Scienze Mediche.* *Ottobre,* 1835.

### *Vaccination in Austria.*

[The following table shows, on a large scale, the state of vaccination in the Austrian dominions, and furnishes some results which cannot be obtained in this country, owing to the imperfect organization of our medical and statistical institutions.]

| Provinces. | Number vaccinated. | No. in which vaccination succeeded. | No. of cases in which vaccination failed. | Number remaining unvaccinated. | Number affected with Smallpox. | |
|---|---|---|---|---|---|---|
| | | | | | Inoculated | Not inocul. |
| 1832. | | | | | | |
| Dalmatia | 9,266 | 9,132 | 134 | 604 | 8 | 28 |
| Hungary | 185,972 | 184,834 | 1,138 | — | — | 1,545 |
| Venetian Territories | 55,222 | 52,740 | 2,482 | 15,416 | 3,981 | 5,706 |
| Austria on the Enns | 19,143 | 18,023 | 1,120 | 20,748 | — | 1,683 |
| 1833. | | | | | | |
| Austria on the Enns | 17,690 | 17,095 | 595 | 18,559 | 177 | 5,289 |
| Moravia and Silesia | 70,302 | 67,500 | 2,802 | 12,400 | 59 | 118 |
| Styria | 20,005 | 19,202 | 803 | 5,331 | — | 1,491 |
| Illyria | 17,119 | — | — | 3,964 | 718 | 2,430 |
| Maritime Territories | 14,432 | 14,260 | 172 | 2,538 | 27 | 133 |

From this table it appears that, of the total number of persons vaccinated, about one in forty-three failed to take the infection; that, of the total population, more than one-fifth remained unvaccinated; that the proportion of persons inoculated with smallpox to those vaccinated was as one to eighty-four; and that the number of cases of natural smallpox to the number of persons vaccinated was as one to twenty-two and a fraction.

*Medizinische Jahrbücher des Oesterr. Staates.* 1835.

---

### *Revaccination in the Prussian Army in* 1834.

[A general revaccination of the military having been ordered in the year 1834, the following are the results, extracted from an official document addressed by the head of the military medical department, Von Wiebel, to the medical officers in the army, and dated Berlin, 23d June, 1835. Like all the medico-statistical documents of Prussia, it is important from its extreme accuracy and the extent of the authentic facts which it embraces.]

1. The number of men vaccinated was 44,454.
2. Of this number,

| | | |
|---|---|---|
| Had distinct marks of previous vaccination | 33,634 | |
| Indistinct marks | 7,134 | |
| No marks | 3,686 | |
| | | 44,454 |

3. The present vaccination was

| | | |
|---|---|---|
| Regular in its course in | 16,679 | |
| Irregular | 12,287 | |
| Without any result | 15,488 | |
| | | 44,454 |

4. Of the number (15,488) in whom the vaccination entirely failed, 4,530 were revaccinated a second time; and, of these, 866 took the disease; while, in the remaining 3,664, no effect was produced.

5. The number of true pustules in the individuals who took the disease regularly was as follows:

| | | | |
|---|---|---|---|
| From | 1 to 5 | in | 6,763 |
| — | 6 to 10 | in | 5,028 |
| — | 11 to 20 | in | 4,088 |
| — | 21 to 30 | in | 800 |
| | | | 16,679 Total. |

6. Of those who had been previously vaccinated, or on the present occasion were revaccinated and took the disease properly, the following were affected with some form of variolous or varioloid disease in the course of the year:

| | |
|---|---|
| With varicella ........ | 46 |
| With varioloid disease.. | 31 |
| With true variola...... | 2 |
| | 79 |

7. In the whole army, the number affected with varicella, varioloid disease, or true smallpox, in the year 1834, were however 619, including the 79 mentioned in the last paragraph; consequently, in 540 of these patients, the revaccination had been either irregular or had not succeeded, or had not been employed at all.

Of these 619 soldiers, 38 died of the true smallpox; of whom, 2 had been vaccinated with effect, 6 without effect, and 30 (nearly all raw recruits) not vaccinated at all.

[From the preceding statements, the medical director very properly urges the advantage of revaccination, and strongly recommends all medical officers to carry into effect once more in all those cases wherein the previous vaccination had been irregular in its course or had entirely failed; instructing them to take the lymph from young subjects, vaccinated for the first time.]

*Rust's Magazin*, Band. xlv. Heft i. 1835.

